# Anterior Inferior Cerebellar Arteries Juxtaposed with the Internal Acoustic Meatus and Their Relationship to the Cranial Nerve VII/VIII Complex

**DOI:** 10.7759/cureus.1570

**Published:** 2017-08-16

**Authors:** Fernando Alonso, Mohammad W Kassem, Joe Iwanaga, Rod J Oskouian, Marios Loukas, Amin Demerdash, R. Shane Tubbs

**Affiliations:** 1 Neurosurgery, University Hospitals of Cleveland, Case Medical Center; 2 Clinical Anatomy Research Fellow, Seattle Science Foundation; 3 Seattle Science Foundation; 4 Swedish Neuroscience Institute; 5 Department of Anatomical Sciences, St. George's University School of Medicine, Grenada, West Indies; 6 Neurosurgery, Seattle Science Foundation

**Keywords:** anterior inferior cerebellar artery, neurosurgery, vertigo, tinnitus, hemifacial spasm

## Abstract

Vascular loops in the cerebellopontine angle (CPA) and their relationship to cranial nerves have been used to explain neurological symptoms. The anterior inferior cerebellar artery (AICA) has variable branches producing vascular loops that can compress the facial cranial nerve (CN) VII and vestibulocochlear (CN VIII) nerves. AICA compression of the facial-vestibulocochlear nerve complex can lead to various clinical presentations, including hemifacial spasm (HFS), tinnitus, and hemiataxia. The formation of arterial loops inside or outside of the internal auditory meatus (IAM) can cause abutment or compression of CN VII and CN VIII.

Twenty-five (50 sides) fresh adult cadavers underwent dissection of the cerebellopontine angle in the supine position. In regard to relationships between the AICA and the nerves of the facial/vestibulocochlear complex, 33 arteries (66%) traveled in a plane between the facial/nervus intermedius nerves and the cochlear and vestibular nerves. Five arteries (10%) traveled below the CN VII/VIII complex, six (12%) traveled posterior to the nerve complex, four (8%) formed a semi-circle around the upper half of the nerve complex, and two (4%) traveled between and partially separated the nervus intermedius and facial nerve proper. Our study found that the majority of AICA will travel in a plane between the facial/nervus intermedius nerves and the cochlear and vestibular nerves. Although the relationship between the AICA and porus acusticus and AICA and the nerves of the CN VII/VIII complex are variable, based on our findings, some themes exist. Surgeons should consider these with approaches to the cerebellopontine angle.

## Introduction

Vascular loops in the cerebellopontine angle (CPA) and their relationship to cranial nerves have been used to explain neurological symptoms. Their effects are most commonly manifested through abutment and compression of the trigeminal and facial nerves, resulting in trigeminal neuralgia and hemifacial spasm, respectively. Vascular compression of the trigeminal nerve leading to trigeminal neuralgia is said to have been first described by Dandy in 1934 [1]. The anterior inferior cerebellar artery (AICA) has variable branches producing vascular loops that can compress the facial cranial nerve (CN) VII and vestibulocochlear (CN VIII) nerves. The possible effects of vascular loops on facial spasm, tinnitus, and diminished hearing have been debated [2-3]. The aim of this study is to identify, localize, and document the relationship between the anterior inferior cerebellar artery (AICA) and the CN VII/VIII complex.

## Materials and methods

Twenty-five (50 sides) fresh adult cadavers underwent dissection of the cerebellopontine angle in the supine position. The mean age at death of the specimens was 69 years (range: 48-95 years). There were 15 male and 10 female specimens. An oscillating bone saw was used to remove the calvaria and then the supratentorial brain was removed. Next, the tentorium cerebelli was carefully cut from its attachment along the petrous part of the temporal bone and folded posteriorly to reveal the CPA. A surgical microscope (OPMI® CS NC31, Carl Zeiss, Oberkochen, Germany) was then used to dissect the CN VII/VIII nerve complex and to localize the AICA. Documentation of the relationship between this artery, the internal acoustic meatus, and its nerves was made. Statistical analysis between sides and gender were performed with statistical significance set at p < 0.05.

## Results

**Figure 1:**
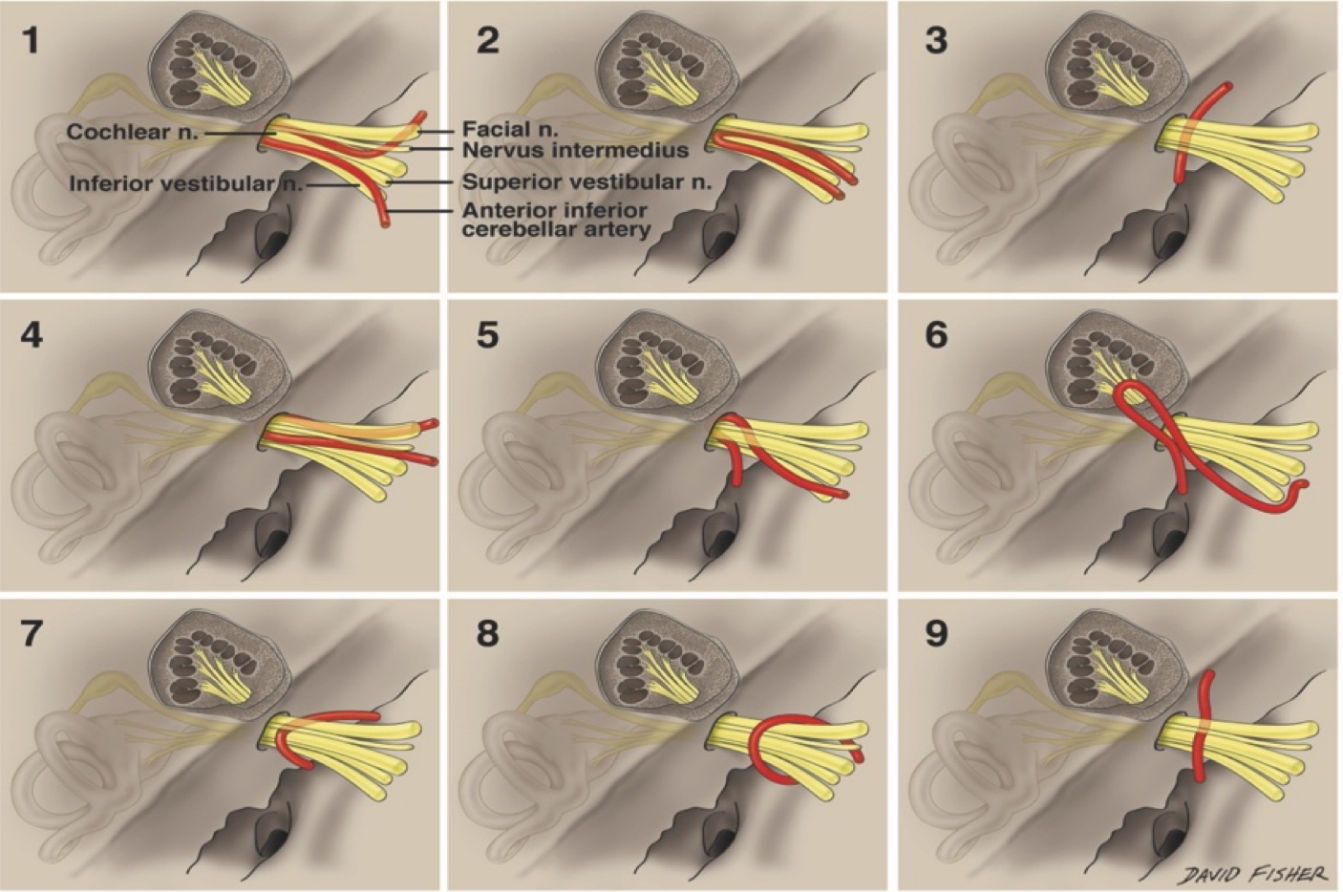
Anterior inferior cerebellar artery in relation to porus acusticus and cranial nerve VII/VIII complex Image 1: AICA (anterior inferior cerebellar artery) and the cranial nerve (CN) VII/VIII complex traveling in a plane between the facial/nervus intermedius nerves and the cochlear and vestibular nerves. AICA at the porus. Image 2: AICA and the CN VII/VIII complex traveling in a plane between the facial/nervus intermedius nerves and the cochlear and vestibular nerves. AICA at the porus. Image 3: AICA traveling below the CN VII/VIII complex. AICA medial to the porus. Image 4: AICA traveling between and partially separated the nervus intermedius and facial nerve proper. AICA at the porus. Image 5: AICA traveling between and partially separated the nervus intermedius and facial nerve proper. AICA at the porus. Image 6: AICA traveling posterior to the nerve complex and extending through the porus and into the internal acoustic meatus. Image 7: AICA and the CN VII/VIII complex traveling in a plane between the facial/nervus intermedius nerves and the cochlear and vestibular nerves, medial to the porus. Image 8: AICA forming a semicircle around the upper half of the nerve complex and medial to the porus. Image 9: AICA and the CN VII/VIII complex traveling in a plane between the facial/nervus intermedius nerves and the cochlear and vestibular nerves, medial to the porus. Original drawings done by David Fisher

An AICA was identified on all sides. A loop of the AICA was found to protrude toward the porus acusticus on 28 sides (56%). Of these vessels that approached the porus, eight (29%) were medial to it (Figure 1, images 3, 7-9), 17 (61%) were at the porus (Figure 1, images 1-2, 4-5), and three (10%) extended through the porus and into the internal acoustic meatus (Figure 1, image 6). In regard to relationships between the AICA and the nerves of the facial/vestibulocochlear complex, 33 arteries (66%) traveled in a plane between the facial/nervus intermedius nerves and the cochlear and vestibular nerves (Figure 1, images 1-2, 7, 9). Five arteries (10%) traveled below the CN VII/VIII complex (Figure 1, image 3), six (12%) traveled posterior to the nerve complex (Figure 1, image 6), four (8%) formed a semicircle around the upper half of the nerve complex (Figure 1, image 8), and two (4%) traveled between and partially separated the nervus intermedius and facial nerve proper (Figure 1, images 4-5). Although a trend was seen with male specimens having arteries that more commonly rested at or within the porus acusticus, this did not reach statistical significance. No statistical significance was found between sides.

## Discussion

Anatomy

The CPA is a V-shaped cleft facing the posterior aspect of the temporal bone. It is bordered by the superior and inferior limbs of the cerebellopontine fissure, which is formed by the folding of the petrosal surface of the cerebellum around the pons and the middle cerebellar peduncle. Eight cranial nerves, CN IV through CN XI, and their origins are located near or within the CPA. The trochlear and trigeminal nerves are close to the superior limb of the cerebellopontine fissure, while the abducens nerve lies at its base. The facial and vestibulocochlear nerves are located at the center of the CPA, and the glossopharyngeal, vagus, and accessory nerves are near the inferior limb of cerebellopontine fissure [4-5].

The neurovascular relationships in the CPA are very significant, and occlusion or sacrifice of vessels of the posterior circulation can lead to various ischemic insults to the brainstem resulting in different stroke patterns. The CPA can be regarded as comprising three neurovascular compartments: superior, middle, and inferior. The superior compartment contains the oculomotor, trochlear, and trigeminal nerves alongside the superior cerebellar artery (SCA), which travels in the cerebello-mesencephalic fissure. As categorized in the present study, the middle compartment contains the AICA within the cerebellopontine fissure in direct relationship with CN VI, VII, and VIII. The inferior compartment contains the posterior inferior cerebellar artery (PICA) in the cerebellomedullary fissure, accompanied by the glossopharyngeal, vagus, and accessory nerves [6].

The AICA originates from the lower or middle third of the basilar artery and supplies the internal auditory meatus and the anteroinferior surface of the cerebellum. It rises as a single artery (72%) or as duplicate (26%) or triplicate (2%) arteries [7]. It can be divided into four segments: anterior pontine, lateral pontine, flocculopeduncular, and cortical. It arises at the level of the pons and travels among CN VI, VII and VIII to reach the middle cerebellar peduncle. It then travels along the cerebellopontine fissure where it ultimately supplies the petrosal surface of the cerebellum [8]. The AICA travels in close proximity to the facial-vestibulocochlear complex, often running between CN VII and CN VIII before delivering its terminal branches. It gives off the labyrinthine artery (LA), which enters the internal auditory meatus alongside the facial and vestibulocochlear nerves [9]. The LA rises from the meatal portion of the AICA in approximately 90% and from the basilar artery in 10% of patients. It courses between CN VII and CN III in 85% or passes over CN VII and CN VIII ventrally. It has a single trunk in 60% of cases and is bi-arterial in 40% [10].

Occlusion of the AICA can result in the lateral pontine syndrome, causing a sudden onset of nausea, vertigo, dysarthria, nystagmus, and injury to the vestibular nuclei, and hence, hemiataxia and injury to the principal sensory trigeminal nucleus. This results in loss of sensation in the face, damage to the facial nucleus, and therefore, facial weakness, hearing loss, and tinnitus due to injury to the cochlear nuclei. Physicians must consider that acute auditory or vestibular compromise can be a precursor to an AICA infarction with resultant occlusion of the basilar artery [11].

Pathology

The close relationship between the AICA, CN VII, CN VIII, and the internal auditory meatus (IAM) has been a subject of discussion concerning artery-nerve conflicts and their resultant symptomatology. AICA compression of the facial-vestibulocochlear nerve complex can lead to various clinical presentations, including hemifacial spasm (HFS), tinnitus, and hemiataxia. The formation of arterial loops inside or outside the IAM can cause abutment or compression of CN VII and CN VIII. Critics of this hypothesis note that arterial loops are common in asymptomatic patients [6, 12]. This has also been observed in patients with trigeminal neuralgia, which is most frequently caused by compression of the trigeminal nerve by the superior cerebellar artery (SCA) [1]. A 17% rate of compression of the trigeminal nerve by the SCA has been reported in asymptomatic patients [6]. Similarly, it has been estimated that vascular compression of CN VII occurs in as many as 53% of asymptomatic patients [13]. McDermott, et al. classified vascular loops of the AICA into three subtypes: Type I, in which an AICA loop occurs within the CP angles; type II, where the AICA loop enters less than half the length of the IAC; and type III, where the AICA enters more than half the length of the IAC [9]. The incidence of AICA vascular loops differs among anatomical and radiological studies. In a cadaveric study, Ouaknine, et al. reported AICA loops in 97% of samples [14]. The relationship of AICA loops to the IAM is a proposed reason for tinnitus resulting from CN VIII compression. Symptoms of vestibulocochlear nerve compression are believed to be caused by direct pulsatile compression with ephaptic discharges [15].

A study of 1,327 temporal bones revealed AICA loops within the IAM in 12.3% of patient samples. The authors noted that most patients complaining of peripheral vertigo had no arterial anomaly compressing the vestibulocochlear complex. Therefore, they concluded that there is no causal link between the auditory symptoms found in a minority of cases and the presence of the AICA in the IAM [9]. When they occur, most AICA loops are found outside the meatus in 50%, reach the orifice of the IAM in 25%, enter the IAM in 19%, and occupy the middle of the IAM in 6% of cases [14].

Most of the literature stating that an abnormal AICA loop can cause compression of CN VIII and lead to auditory symptoms is based on case reports. Compression of the CN VII root exit zone (REZ) is a recognized cause of hemifacial spasm (HFS). The REZ is referred to as the Obersteiner-Redlich zone, which demarcates the transition between central and peripheral axon myelination as the nerve detaches from the pons. Nagahiro, et al. identified seven patients with a meatal branch of the AICA compressing the dorsal aspect of CN VII, causing hemifacial spasm with a poor postoperative outcome. The meatal segment of the AICA often crosses the gap between CN VII and VIII and can compress the cisternal portion of CN VII. Prominent loops of the AICA have been implicated in the compression of CN VIII. Applebaum, et al. tested the audiometric and vestibular system in 15 patients with vascular loops in the internal auditory canal (IAC). They concluded that a cochlear type hearing loss with good speech discrimination and normal caloric testing should raise the suspicion of a vascular loop compressing CN VIII [16].

A study of 72 patients who underwent microvascular decompression for severe tinnitus and conduction abnormalities of the auditory nerve showed that 18.2% experienced total tinnitus relief, 22.2% had significant improvement, 11% minimal improvement, and 45.8% with no improvement, and with 2.8% becoming worse. The operation was the most successful for patients who had been symptomatic for less than three years, so it can be assumed that continued compression would lead to persistent nerve damage. A series of 207 patients who underwent microvascular decompression for disabling positional vertigo revealed that 79% were free of symptoms or markedly improved postoperatively [17].

A review of the literature identified 35 studies of neurovascular compression of the cochlear-vestibular nerve by the AICA in a total of 536 patients. Only nine of these patients were identified as having intrameatal AICA nerve-vascular conflicts. All nine suffered from tinnitus and, of these, two from vertigo and one from unilateral hearing loss. Tinnitus and vertigo resolved after surgery but the hearing loss did not improve [18]. Hearing loss could, therefore, be a sign of more chronic compression leading to irreversible nerve injury. The study concluded that patients with intrameatal compression should be offered microvascular decompression prior to the development of hearing loss. Ezerarslan, et al. found that a type IIB branching pattern is more common in patients with an idiopathic sudden sensorineural hearing loss, and unlike non-type IIB, branching patterns are associated with unresponsiveness to standard oral corticosteroid therapy [19].

A blinded analysis of 167 magnetic resonance imaging (MRI) scans of the CP angle found an AICA loop in 94% of patients. Of the loop types found, 196 were type I, 106 were type II, and 14 were type III. Sixty-six of those patients had an unexplained unilateral hearing loss. However, there was no association between the presence of type II or type III vascular loop, IAC width, and unilateral hearing loss, further supporting the view that AICA loop extension depth into the IAC does not correlate with unilateral hearing loss [20]. Makins, et al. obtained similar results from their study of 112 patients with idiopathic unilateral auditory symptoms. That study used the asymptomatic contralateral ears as controls and found no significant difference in the arterial loop contact with CN VIII between the symptomatic and asymptomatic sides [21].

Advances in imaging techniques have led to a more effective identification of vessel-nerve compromise. Three-dimensional (3D) MRI reportedly has a very high sensitivity, with only one false negative result [22]. Both 1.5-T and 3.0-T surface-rendered 3D imaging provide high sensitivity and specificity for preoperative identification of trigeminal neuralgia compression by the SCA. However, 3.0-T MRI is superior for identifying smaller vessels, such as the AICA [23].

Casselman, et al. were the first to use three-dimensional Fourier-transform constructive interference in steady state (3D FT-CISS) for inner ear and CP angle imaging; it has since been commonly used for identifying CN compression [24-25]. Raybaud, et al. used constructive interference in steady state (CISS) imaging on several patients with either CN VII or CN VIII deficits and compared their results to a control cohort. They found that the AICA caused nerve compression in the IAM in each of the five patients who presented with tinnitus, while only 5% of the asymptomatic control group had a nerve-artery conflict [26]. A study correlating cadaveric with MRI findings concluded that 3D-FT-CISS MRI provided better imaging than 3D time-of-flight (TOF) magnetic resonance angiography (MRA) for analyzing the relationship between the AICA and the facial-vestibulocochlear nerve complex [27]. Three-dimensional fast imaging employing steady-state acquisition (3D FIESTA) MRI can be used to obtain detailed thin section images with high spatial resolution so that the cranial nerves can be depicted clearly within the cisternal spaces [19].

## Conclusions

Although the relationships between the AICA and porus acusticus and AICA and the nerves of the VII/VIII complex are variable, based on our findings, some themes do exist. Our study found that the majority of AICA will travel in a plane between the facial/nervus intermedius nerves and the cochlear and vestibular nerves. Surgeons should consider these with approaches to the CPA.
